# Expression Patterns and Correlations with Metabolic Markers of Zinc Transporters *ZIP14* and *ZNT1* in Obesity and Polycystic Ovary Syndrome

**DOI:** 10.3389/fendo.2017.00038

**Published:** 2017-03-02

**Authors:** Trine Maxel, Pernille Fog Svendsen, Kamille Smidt, Jesper Krogh Lauridsen, Birgitte Brock, Steen Bønlykke Pedersen, Jørgen Rungby, Agnete Larsen

**Affiliations:** ^1^Faculty of Health, Department of Biomedicine, Aarhus University, Aarhus, Denmark; ^2^Department of Obstetrics and Gynecology, Herlev University Hospital, Herlev, Denmark; ^3^Faculty of Health, Department of Clinical Medicine, Aarhus University, Aarhus, Denmark; ^4^Department of Clinical Biochemistry, Aarhus University Hospital, Aarhus, Denmark; ^5^Department of Endocrinology (MEA), Aarhus University Hospital, Aarhus, Denmark; ^6^Center for Diabetes Research, Department of Medicine, Gentofte University Hospital, Hellerup, Denmark

**Keywords:** obesity, polycystic ovary syndrome, peroxisome proliferator-activated receptor gamma, Slc30a, Slc39a, ZIP14, *ZNT1*, zinc

## Abstract

Polycystic ovary syndrome (PCOS) is associated with infertility, increased androgen levels, and insulin resistance. In adipose tissue, zinc facilitates insulin signaling. Circulating zinc levels are altered in obesity, diabetes, and PCOS; and zinc supplementation can ameliorate metabolic disturbances in PCOS. In adipose tissue, expression of zinc influx transporter *ZIP14* varies with body mass index (BMI), clinical markers of metabolic syndrome, and *peroxisome proliferator-activated receptor gamma* (*PPARG*). In this study, we investigated expression levels of *ZIP14* and *PPARG* in subcutaneous adipose tissue of 36 PCOS women (17 lean and 19 obese women) compared with 23 healthy controls (7 lean and 16 obese women). Further, expression levels of zinc transporter *ZIP9*, a recently identified androgen receptor, and zinc efflux transporter *ZNT1* were investigated, alongside lipid profile and markers of glucose metabolism [insulin degrading enzyme, retinol-binding protein 4 (*RBP4*), and glucose transporter 4 (*GLUT4*)]. We find that *ZIP14* expression is reduced in obesity and positively correlates with *PPARG* expression, which is downregulated with increasing BMI. *ZNT1* is upregulated in obesity, and both *ZIP14* and *ZNT1* expression significantly correlates with clinical markers of altered glucose metabolism. In addition, *RBP4* and *GLUT4* associate with obesity, but an association with PCOS as such was present only for *PPARG* and *RBP4*. *ZIP14* and *ZNT1* does not relate to clinical androgen status and *ZIP9* is unaffected by all parameters investigated. In conclusion, our findings support the existence of a zinc dyshomeostasis in adipose tissue in metabolic disturbances including PCOS-related obesity.

## Introduction

Polycystic ovary syndrome (PCOS) is the most common cause of infertility, affecting approximately 12−18% of all women of reproductive age (1). The syndrome is linked to increased risk of cardiovascular disease and type 2 diabetes (2). High androgen levels and oligo/anovulation are characteristics of PCOS, as are metabolic disturbances such as insulin resistance, visceral obesity, and dyslipidemia (2).

Altered serum-zinc levels are found in women with PCOS compared to body weight-matched, healthy controls, and both increased and decreased zinc levels have been reported (3–5). Interestingly, 8 weeks of zinc supplementation has beneficial effects on fasting plasma glucose, insulin resistance as measured by homeostatic model assessment (HOMA index), and the lipid profile in PCOS women (6). Similar to PCOS, a disturbed zinc homeostasis is also common in type 2 diabetic and obese individuals, which is evident by the presence of hypozincemia (7–10). Low zinc intake in obese individuals has been linked to hyperinsulinemia, increased low-grade inflammation, and an aggravated lipid profile (11). These results indicate the presence of a disturbed zinc homeostasis in PCOS women, potentially linked with their obesity, and with an adverse effect on their metabolic health.

The essential trace metal zinc is required for the function of more than 300 enzymes and with an effect on at least 2,000 transcription factors (12). Free cytoplasmic zinc also regulates gene expression through the metal-responsive transcription factor-1 (13) and the level of free zinc can affect intracellular signaling pathways, e.g., by inhibition of protein tyrosine phosphatase (14). Due to its intracellular signaling and structural functions, zinc plays a role in lipid and glucose metabolism as well as in fertility (12, 15). Zinc can activate the insulin signaling pathway through inhibition of protein tyrosine phosphatase leading to increased phosphorylation of the insulin receptor (14). Further, zinc ions can work in an insulinomimetic way within adipocytes, stimulating lipogenesis and glucose transport through translocation of glucose transporter 4 (GLUT4) to the cell membrane (16, 17). In the reproductive system, zinc deficiency associates with poor pregnancy outcomes and zinc is crucial for normal sexual development in both men and women (12, 18–21). Animal studies show that zinc deficiency affects ovulation, as well as zinc has an effect on the estrogen level and menstrual cycle (22, 23). In men, zinc deficiency is linked to decreased circulating levels of testosterone and dihydrotestosterone (DHT) (23).

The regulation of intracellular zinc homeostasis is maintained by a number of zinc transporters and metallothioneins (24, 25). Fourteen human zinc influx transporters (SLC39a; ZIP1–14) and 10 zinc-efflux transporters (SLC30a; *ZNT1–10*) have so far been identified (24, 25).

The zinc influx transporter ZIP14 is a member of the LIV-1 subfamily of zinc transporters that localize to the plasma membrane and facilitate the transport of zinc into the cell (26). ZIP14 is believed to be important in adipogenesis (27–29). We recently reported that *ZIP14* expression in adipose tissue is reversibly downregulated by obesity and inversely correlated with clinical markers of metabolic disease (27). Further, we found a possible link between *ZIP14* expression and the transcription factor *peroxisome proliferator-activated receptor gamma* (*PPARG*) in humans (27), a finding also seen in studies of adipose tissue from Zip14 knockout mice (29). PPARG regulates lipid uptake and glucose metabolism, and plays an essential role in adipogenesis (30). Polymorphisms in the PPARG gene have been shown to decrease the risk and affect the metabolic phenotype of PCOS (31, 32) and the function of PPARG is regulated by zinc status (33, 34). Similar to other members of the LIV-1 subfamily of zinc transporters, estrogen can induce zinc influx through ZIP14 (26, 35, 36) and another zinc influx transporter, ZIP9, has recently been identified as an androgen receptor in human hormone-responsive breast and prostate cancer cells, with androgens stimulating ZIP9-mediated zinc influx (37, 38).

In this study, we aimed at investigating if intracellular zinc dyshomeostasis, as reflected by changes in expression of zinc transporters, is directly linked to the PCOS syndrome. Specifically, we aimed at investigating the expression levels of *ZIP14*, due to its indicated role in adipose tissue functioning and association with metabolic disease. A potential sex-hormonal and metabolic regulation of *ZIP14*, as a member of the LIV-1 subfamily, were investigated together with the expression levels of the androgen receptor *ZIP9* and the main zinc efflux transporter *ZNT1*, in adipose tissue from lean and obese women with PCOS as well as weight-matched controls. The expression levels were correlated with clinical markers of lipid and glucose metabolism, androgen status, as well as expression of genes related to glucose metabolism within adipose tissue, i.e., *GLUT4, insulin degrading enzyme* (*IDE*), and *retinol-binding protein 4* (*RBP4*), all speculated to be involved in metabolic disease.

## Materials and Methods

### Participants and Interventions

Thirty-six lean [body mass index (BMI) = 18.5–25; *n* = 17] and obese (BMI > 25; *n* = 19) women with PCOS, and 23 age-matched lean (*n* = 7) and obese (*n* = 16) women without PCOS (control), recruited through advertising in the local newspaper, were included in the study. The women in the control group had a regular menstrual cycle (<35 days) and an androgen level within the normal range. None of the participants suffered from other chronic diseases or used oral contraceptives or any other drugs known to alter glucose or insulin metabolism within the last 3 months prior to the study. PCOS was diagnosed according to the Rotterdam criteria (39), i.e., based on the androgen level, evaluation of hirsutism (Ferriman–Gallwey score ≥8), transvaginal ultrasonography, and menstrual history (40). The study design and assessment of clinical parameters (PCOS status, anthropometrics, insulin and glucose measurements, lipid profile, and hormone analysis) have been previously described (40–44). The study was performed at the Department of Endocrinology and Department of Obstetrics and Gynecology, Hvidovre University Hospital, Hvidovre, Denmark. The study was approved by the local ethics authorities and followed the principles in the “Declaration of Helsinki.” All the subjects gave their written informed consent prior to the study.

### Determination of Body Fat Percentage and Measurement of Glucose Metabolism, Lipid Profile, and Sex Hormones

Participants underwent a dual-emission X-ray absorptiometry scan to determine the body fat percentage. Subcutaneous fat biopsies for gene expression studies were obtained from the lower abdomen. Blood samples were collected following an overnight fasting. Plasma glucose was measured using a Beckman Glucose analyzer (Ramcon, Fullerton, CA, USA), and serum insulin was determined using the 1235 Auto DELPHIA Automatic Immunoassay System (Wallac Oy, Turku, Finland). The HOMA index was calculated as follows: fasting serum insulin concentration (μU/L) × fasting plasma glucose concentration (mmol/L)/22.5 (45, 46). Fasting levels of cholesterol, high-density lipoprotein (HDL), low-density lipoprotein, very-low-density lipoprotein (VLDL), and triglycerides were measured by reflection photometry (Ortho-Clinical diagnostics kit, Raritan, NJ, USA). Testosterone, DHT, sex hormone-binding globulin (SHBG), and the calculation of free testosterone were performed as previously described using immunofluorometric assays and radioimmunoassays (40).

### Real-time Polymerase Chain Reaction (PCR)

RNA was extracted from the fat biopsies using TRIzol (Gibco BRL, Life Technologies, Roskilde, Denmark). The concentration and quality of the purified total RNA were determined spectrophotometrically and by running an agarose gel. Reverse transcription was performed using the Thermo Scientific Verso cDNA kit (Applied Biosystems, Foster City, CA, USA). The KAPA SYBR FAST qPCR kit (Kapa Biosystems, Inc., Woburn, MA, USA) and a LightCycler 480 (Roche Applied Science, Basel, Switzerland) were used for the real-time PCRs. Duplicate reactions of all samples were performed. The expression levels of *ZIP14, ZIP9, ZNT1, PPARG1, PPARG2, IDE, RBP4*, and *GLUT4* were measured. Gene accession numbers and primers are listed in Table S1 in Supplementary Material. *Low-density lipoprotein receptor-related protein-10* (*LRP10*) was used as a housekeeping gene, as it shows a stable expression in human adipose tissue (47). The stability of *LRP10* was confirmed by statistically comparing *C*_t_-cycles between groups. Relative expression levels were calculated using the Advanced Relative Quantification mode of the LightCycler 480 instrument, version 1.5.0.39 (Roche Applied Science).

### Statistical Analysis

Metabolic data and androgen status are presented as the means ± SD, and the groups were compared by Student’s *t*-test. Data were found to be normally distributed (QQ plots), except VLDL and HOMA index values which were log-transformed to obtain a normal distribution. Real-time PCR data are presented as the mean starting quantity of (gene of interest/*LRP10*) ± SEM. Statistical analyses of the PCR results were performed on log-transformed data to obtain a normal distribution. The influence of PCOS and obesity and their interaction with regards to the expression of the genes were examined by two-way ANOVA analysis. In the presence of a statistically detectable interaction, a combined (interfering) effect exists in which the relationship between a factor, e.g., weight status and the effect on gene expression differs by the presence of the other factor, e.g., PCOS. If statistically significant differences were found among the four groups, subgroup analyses were performed using Student’s *t*-test in order to identify the origins of this difference; e.g., following a significant two-way ANOVA showing an effect of obesity, obese PCOS women vs. lean PCOS women, and obese control women vs. lean control women were compared. Correlations included the whole study population (*n* = 59) and were performed using a standard Pearson’s correlation and log-transformed PCR data. Although specifically for *PPARG1*, correlations were additionally done separately for PCOS and non-PCOS women, as *PPARG1* showed a regulation by weight and PCOS status. The level of statistical significance in all analyses was set at 0.05. STATA (StataCorp LP, TX, USA) and GraphPad Prism 5 (GraphPad Software Inc., La Jolla, CA, USA) statistical packages were used for the calculations.

## Results

### Metabolic Measurements and Androgen Status of PCOS Women and Controls

Metabolic and androgen measurements were performed on all subjects to establish their metabolic status and to confirm the presence or absence of PCOS pathology.

Anthropometric findings, insulin and glucose measurements, the lipid profile, and the results of the hormone analysis are shown in Table 1. For further details on the metabolic and sex-hormonal measurements, please refer to Ref. (40, 41, 43, 44). The age was comparable between groups (40). In addition, BMI was similar between lean PCOS women and lean controls, and between obese PCOS women and obese controls (*p* = 0.1707 and *p* = 0.2482, respectively). As expected, the HOMA index was significantly higher in the obese women than that in lean women in both the PCOS and control groups (*p* = 0.0002 and *p* < 0.0001, respectively). The total testosterone level was significantly increased in the PCOS women compared to the control women in both the lean and obese groups (*p* = 0.01 and *p* < 0.0001, respectively) (40, 41, 43, 44).

**Table 1:** **Metabolic measurements and androgen status of lean and obese women with PCOS, and lean and obese control women without PCOS**.

	PCOS		Controls	
	Lean PCOS (*n* = 17)	Obese PCOS (*n* = 19)	Lean controls (*n* = 7)	Obese controls (*n* = 16)
Age (years)	28 (±5)	29 (±4)	30 (±4)	31 (±5)
BMI (kg/m^2^)	23 (±2)^●●●^	33 (±4)	22 (±2)^●●●^	34 (±3)
Body fat (%)	27 (±4)^●●●^	41 (±7)	24 (± 7)^●●●^	43 (±6)
Insulin (pmol/l)	36 (±18)^●●^	67 (±39)	25 (±14)^●●^	64 (±31)
Glucose (mmol/l)	5.5 (±0.4)^●^	6.0 (±0.6)^○^	5.6 (±0.4)	5.6 (±0.3)
HOMA-IR	1.5 (±0.8)^●●●^	3.0 (±1.8)	1.0 (±0.6)^●●●^	2.7 (±1.3)
HDL (mmol/l)	1.4 (±0.3)^○,●●●^	1.1 (±0.2)	1.1 (±0.2)	1.1 (±0.2)
VLDL (mmol/l)	0.4 (±0.2)	0.4 (±0.2)	0.4 (±0.1)	0.6 (±0.3)
Cholesterol (mmol/l)	4.3 (±1.0)	4.2 (±0.6)	3.6 (±0.6)^●●^	4.7 (±0.8)
Triglycerides (mmol/l)	0.8 (±0.4)	1.0 (±0.3)	0.8 (±0.2)^●^	1.2 (±0.6)
T-testosterone (nmol/l)	2.1 (±0.8)^○^	2.4 (±0.8)^○○○^	1.4 (±0.3)	1.4 (±0.4)
F-testosterone (nmol/l)	0.034 (±0.019)^○○^	0.043 (±0.022)^○○^	0.015 (±0.006)^●^	0.024 (±0.007)
SHBG (nmol/l)	67 (±27)^○^	59 (±39)	101 (±38)^●●●^	54 (±21)

*Data are presented as the means ± SD*.

*BMI, body mass index; F-testosterone, free testosterone; HDL, high-density lipoprotein; HOMA-IR, homeostatic model assessment-insulin resistance; PCOS, polycystic ovary syndrome; SHBG, sex hormone-binding globulin; T-testosterone, total testosterone; VLDL, very-low-density lipoprotein*.

*^○^p < 0.05, ^○○^p < 0.01, ^○○○^p < 0.001 comparing lean PCOS subjects vs. lean controls or obese PCOS subjects vs. obese controls using Student’s t-test*.

*^●^p < 0.05, ^●●^p < 0.01, ^●●●^p < 0.001 comparing lean PCOS subjects vs. obese PCOS subjects or lean controls vs. obese controls*.

*Level of statistical significance: 0.05*.

In summary, we confirmed the expected differences in BMI, HOMA index, and androgen levels of the four groups (lean and obese women with and without PCOS), establishing that age did not differ significantly among groups nor did BMI differ between the obese controls and the obese PCOS women or between lean controls and lean PCOS women.

### *ZIP14* and *ZNT1* Expression Are Oppositely Regulated in Obesity but Appear Unaffected by PCOS Status, While *ZIP9* Expression in Adipose Tissue Is Unaffected in Both Obesity and PCOS

Adipose expression levels of *ZIP14, ZIP9*, and Z*NT1* were investigated in order to identify if their gene expression is regulated by obesity and/or PCOS status, as we have previously reported a regulation of obesity on adipose *ZIP14* expression (27).

**Figure 1:**
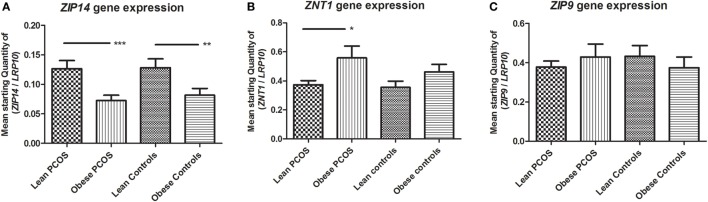
**Expression of zinc transporters *ZIP14, ZNT1*, and *ZIP9* in adipose tissue from lean and obese women with polycystic ovary syndrome (PCOS), and from lean and obese control women without PCOS**. Results are expressed as the mean starting quantity of (gene of interest/*LRP10*) ± SEM. **(A)** Expression of *ZIP14*. **(B)** Expression of *ZNT1*. **(C)** Expression of *ZIP9*. *p < 0.05; **p < 0.01; ***p < 0.001. LRP10, low-density lipoprotein receptor-related protein-10. Lean PCOS women, *n* = 17; obese PCOS women, *n* = 19; lean control women, *n* = 7; obese control women, *n* = 16. The groups were compared by two-way ANOVA analysis followed by subgroup analysis by Student’s *t*-test if significant. Level of statistical significance: 0.05.

Zinc influx transporter *ZIP14* and zinc efflux transporter *ZNT1* expression were oppositely regulated in obese women. *ZIP14* expression decreased significantly in obese individuals compared with lean individuals by two-way ANOVA analysis (*p* < 0.0001; subgroup analysis examining lean PCOS vs. obese PCOS, and lean controls vs. obese controls using Student’s *t*-test; *p* = 0.0008 and *p* = 0.0079, respectively). Neither PCOS status (*p* = 0.3892) nor any combinatory influence of PCOS and weight status had a significant effect on *ZIP14* expression by two-way ANOVA analysis (*p* = 0.6908) (Figure 1A).

Obesity showed a significant upregulatory effect on *ZNT1* expression by two-way ANOVA analysis (*p* = 0.0177; subgroup analysis examining lean PCOS vs. obese PCOS, and lean controls vs. obese controls using Student’s *t*-test; *p* = 0.0169 and *p* = 0.2489, respectively). However, PCOS status did not relate to *ZNT1* expression, and no combinatory effect between PCOS status and weight was found with regards to *ZNT1* expression levels using two-way ANOVA analysis (*p* = 0.3577 and *p* = 0.6396, respectively) (Figure 1B).

*ZIP9* expression was unaffected by both obesity and PCOS status, and no combinatory effect between the two parameters was seen by two-way ANOVA analysis (*p* = 0.4764, *p* = 0.8036, and *p* = 0.3516, respectively) (Figure 1C).

In conclusion, *ZIP14* and *ZNT1* were oppositely regulated by obesity, with a downregulation of *ZIP14* and upregulation of *ZNT1*, but the adipose expression of these zinc transporters was not significantly altered by PCOS status.

### *PPARG1* Expression in Adipose Tissue Is Downregulated by Both Obesity and PCOS

With a known function in lipid and glucose metabolism as well as a role in adipogenesis, adipose *PPARG* expression levels (both isoforms 1 and 2) were investigated in terms of a potential regulation of these genes by PCOS status alone or in combination with obesity.

**Figure 2:**
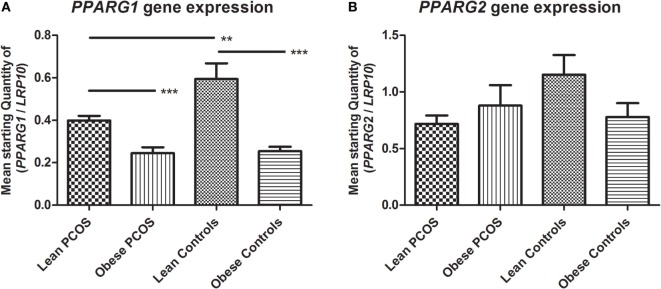
**Expression of peroxisome proliferator-activated receptor gamma (PPARG) in adipose tissue from lean and obese women with polycystic ovary syndrome (PCOS), and from lean and obese control women without PCOS**. Results are expressed as the mean starting quantity of (gene of interest/*LRP10*) ± SEM. **(A)** Expression of *PPARG1*. **(B)** Expression of *PPARG2*. **p < 0.01; ***p < 0.001. LRP10, low-density lipoprotein receptor-related protein-10; PPARG1, peroxisome proliferator-activated receptor gamma mRNA isoform 1; PPARG2, peroxisome proliferator-activated receptor gamma mRNA isoform 2. Lean PCOS women, *n* = 17; obese PCOS women, *n* = 19; lean control women, *n* = 7; obese control women, *n* = 16. The groups were compared by two-way ANOVA analysis followed by subgroup analysis by Student’s *t*-test if significant. Level of statistical significance: 0.05.

The mRNA isoforms *PPARG1* and *PPARG2* were investigated. Two-way ANOVA analysis showed a significant downregulation of *PPARG1* expression by obesity (*p* < 0.0001; subgroup analysis examining lean PCOS vs. obese PCOS, and lean controls vs. obese controls using Student’s *t*-test; *p* = 0.0001 and *p* < 0.0001, respectively). In addition, two-way ANOVA testing showed a significant downregulatory effect of PCOS on *PPARG1* expression (*p* = 0.0291; subgroup analysis examining lean PCOS vs. lean controls, and obese PCOS vs. obese controls using Student’s *t*-test; *p* = 0.0045 and *p* = 0.5380, respectively). However, no additional combined effect of PCOS and weight status was found with regards to *PPARG1* expression (*p* = 0.1739) (Figure 2A). Two-way ANOVA showed no significant effect of weight or PCOS status, nor any combined effect of the two on *PPARG2* expression (*p* = 0.2021, *p* = 0.1244, and *p* = 0.0701, respectively). Notably, mean expression levels were highest in lean controls, as found for *PPARG1* (Figure 2B).

In summary, the expression of *PPARG1* was significantly downregulated by the presence of PCOS as well as by the presence of obesity. However, the combined effect of PCOS and obesity did not statistically differ from the presence of just one of these conditions. Statistically, *PPARG2* showed no regulation by PCOS or obesity status, but showed a similar expression pattern to that of *PPARG1* with a high expression in lean controls compared to the other groups, indicating a potential negative effect of PCOS and obesity.

### Expression of *ZIP14* in Adipose Tissue Is Correlated with *PPARG*, BMI, and Body Fat Percentage

As *ZIP14* expression levels were found to be regulated by obesity and ZIP14 is believed to play a role in adipogenesis, the association with the measured metabolic markers was investigated in terms of the correlation between *ZIP14* expression and anthropometric measurements, lipid profile, and adipogenesis marker *PPARG* using Pearson’s correlation.

*ZIP14* expression associated closely with that of *PPARG*, with a significant positive correlation between the two genes, for both *PPARG1* and *PPARG2* levels (*p* = 0.0001, and *p* = 0.0046, respectively). When investigating the correlation between *PPARG1* and *ZIP14* expression separately for PCOS and non-PCOS women, as *PPARG1* was shown to be statistically independently affected by PCOS status, similar correlations were found (PCOS: *r* = 0.49, *p* = 0.0022; non-PCOS: *r* = 0.48, *p* = 0.0210).

*ZIP14* expression showed a strong inverse correlation with the anthropometric markers BMI (*p* = 0.0001) and body fat percentage (*p* = 0.0027). With regard to the lipid profile, *ZIP14* expression showed a positive correlation with the HDL level (*p* = 0.0421), but no significant correlations were found with other lipid profile markers (VLDL, triglyceride, or cholesterol levels). All *r*-values and *p*-values are shown in Table 2.

**Table 2:** **Correlations among *ZIP14* expression and anthropometric parameters, markers of lipid and glucose homeostasis, and markers of androgen function**.

	*ZIP14* expression
*PPARG1*	*r* = 0.48, *p* = 0.0001
*PPARG2*	*r* = 0.36, *p* = 0.0046
*BMI*	*r* = −0.47, *p* = 0.0001
Body fat percentage	*r* = −0.39, *p* = 0.0027
Insulin	*r* = −0.09, *p* = 0.4955
Glucose	*r* = −0.32, *p* = 0.0120
HOMA-IR	*r* = −0.23, *p* = 0.0822
HDL	*r* = 0.27, *p* = 0.0421
VLDL	*r* = 0.01, *p* = 0.9238
Triglyceride	*r* = −0.00, *p* = 0.9748
Cholesterol	*r* = −0.02, *p* = 0.8888
T-testosterone	*r* = −0.10, *p* = 0.4617
F-testosterone	*r* = −0.34, *p* = 0.1070
SHBG	*r* = 0.16, *p* = 0.2169
Ferriman–Gallwey	*r* = −0.14, *p* = 0.2849
*RBP4* expression	*r* = 0.33, *p* = 0.0117
*GLUT4* expression	*r* = 0.42, *p* = 0.0009

*All women were included (*n* = 59) in this analysis. Pearson’s *r*-values and *p*-values are indicated. Level of statistical significance: 0.05*.

*BMI, body mass index; F-testosterone, free testosterone; GLUT4, glucose transporter 4; HDL, high-density lipoprotein; HOMA-IR, homeostatic model assessment-insulin resistance; PPARG, peroxisome proliferator-activated receptor gamma; RBP4, retinol-binding protein 4; SHBG, sex hormone-binding globulin; T-testosterone, total testosterone; and VLDL, very-low-density lipoprotein*.

In summary, *ZIP14* showed a close inverse relationship with anthropometric markers of obesity as well as a positive association with the adipose expression of *PPARG*.

### *ZIP14* Expression in Adipose Tissue Is Significantly Correlated with Several Markers of Glucose Metabolism in Blood As Well As in Adipose Tissue, but Not with PCOS Markers

As *ZIP14* expression was found to be regulated by obesity, in which alterations in glucose homeostasis are found, we tested a potential association between *ZIP14* expression and glucose homeostatic markers. Further we aimed at confirming that *ZIP14* expression levels do not associate with PCOS status by investigating the association between *ZIP14* expression and specific PCOS markers.

With regard to glucose metabolism, *ZIP14* inversely correlated with fasting glucose levels (*p* = 0.0120). However, *ZIP14* showed no significant correlation with fasting insulin levels or the HOMA index; although in the latter case, the correlation did approach significance (*p* = 0.0822). In adipose tissue, *ZIP14* expression showed significant positive correlations with the glucose metabolic markers, *RBP4* (*p* = 0.0117) and *GLUT4* (*p* = 0.0009). *ZIP14* expression showed no correlation with the PCOS indicators, namely, testosterone, SHBG, or the Ferriman–Gallwey score of hirsutism. All *r*-values and *p*-values are shown in Table 2.

In short, using a Pearson’s correlation, *ZIP14* showed an association with glucose homeostatic markers in adipose tissue and fasting glucose levels. No association was found with specific PCOS markers, confirming the statistical findings of the two-way ANOVA analysis.

### *ZNT1* Showed Associations That Were Opposite to Those of *ZIP14* with BMI and Body Fat Percentage

As the main zinc efflux transporter at the cell membrane, and due to its opposite regulation than *ZIP14* by obesity, we investigated if *ZNT1* expression was associated with the same parameters as *ZIP14*, in terms of anthropometric markers, lipid profile, and *PPARG* levels.

Using Pearson’s correlation, *ZNT1* showed associations that were opposite to those of *ZIP14* with the measured anthropometric markers, as *ZNT1* expression displayed a significant positive correlation with BMI (*p* = 0.0199) and body fat percentage (*p* = 0.0140). However, *ZNT1* expression did not associate with the two *PPARG* isoforms (*PPARG1, p* = 0.2311; *PPARG2, p* = 0.1826), although when analyzing non-PCOS and PCOS women separately, as for *ZIP14*, a significant correlation with *PPARG1* was observed in PCOS women (*r* = −0.34, *p* = 0.0401) but not in non-PCOS women (*r* = 0.13, *p* = 0.5685). The correlation of *ZNT1* expression with the lipid profile showed a negative association with HDL (*p* = 0.0468). No other significant correlations were found. All *r*-values and *p*-values are shown in Table 3.

**Table 3:** **Correlations among *ZNT1* expression and anthropometric parameters, markers of lipid and glucose homeostasis, and markers of androgen function**.

	*ZNT1* expression
*PPARG1*	*r* = −0.16, *p* = 0.2311
*PPARG2*	*r* = 0.18, *p* = 0.1826
*BMI*	*r* = 0.30, *p* = 0.0199
Body fat percentage	*r* = 0.32, *p* = 0.0140
Insulin	*r* = 0.29, *p* = 0.0277
Glucose	*r* = 0.16, *p* = 0.2268
HOMA-IR	*r* = 0.30, *p* = 0.0213
HDL	*r* = −0.26, *p* = 0.0468
VLDL	*r* = 0.10, *p* = 0.4634
Triglyceride	*r* = 0.15, *p* = 0.2423
Cholesterol	*r* = −0.01, *p* = 0.9125
T-testosterone	*r* = 0.15, *p* = 0.2423
F-testosterone	*r* = 0.03, *p* = 0.8006
SHBG	*r* = 0.02, *p* = 0.8886
Ferriman–Gallwey	*r* = 0.10, *p* = 0.4377
*RBP4* expression	*r* = 0.01, *p* = 0.9437
*GLUT4* expression	*r* = 0.05, *p* = 0.6917

*All women (*n* = 59) were included in the analysis. Pearson’s *r*-values and *p*-values are indicated. Level of statistical significance: 0.05*.

*BMI, body mass index; F-testosterone, free testosterone; GLUT4, glucose transporter 4; HDL, high-density lipoprotein; HOMA-IR, homeostatic model assessment-insulin resistance; PPARG, peroxisome proliferator-activated receptor gamma; RBP4, retinol-binding protein 4; SHBG, sex hormone-binding globulin; T-testosterone, total testosterone; and VLDL, very-low-density lipoprotein*.

In summary, *ZNT1* showed an opposite association, to that of *ZIP14*, in terms of the anthropometric markers, but with no general association to *PPARG* expression levels, aside from the subgroup analysis of PCOS women only.

### Markers of Glucose Homeostasis Significantly Correlated with *ZNT1* Expression

As *ZNT1* expression showed an opposite regulation by obesity than that of *ZIP14*, expression levels of *ZNT1* were correlated with glucose homeostasis and markers specifically associated with PCOS, to investigate if *ZNT1* associated with the same parameters as *ZIP14* using a Pearson’s correlation.

Of the markers of glucose homeostasis in the blood and adipose tissue, *ZNT1* showed a significant positive correlation with the fasting insulin level (*p* = 0.0277) and the HOMA index (*p* = 0.0213). There was no statistical significant correlation of *ZNT1* expression with fasting glucose levels or *RBP4* and *GLUT4* expression in adipose tissue. *ZNT1* showed no statistical significant correlations with PCOS indicators, namely, testosterone, SHBG, or the Ferriman–Gallwey score of hirsutism. All *r*-values and *p*-values are shown in Table 3.

In summary, *ZNT1* showed a correlation with blood glucose homeostatic markers, rather than markers of glucose homeostasis in adipose tissue. Confirming the results of the two-way ANOVA analysis, no association of *ZNT1* expression was found with PCOS markers.

### *RBP4* and *GLUT4* Expression Is Downregulated in Adipose Tissue in Obese Women, with PCOS Status Affecting the *RBP4* Level, While *IDE* Expression Is Unaffected by Obesity and PCOS Status

As markers of glucose homeostasis within adipose tissue, *RBP4, GLUT4*, and *IDE* are all associated with the presence of insulin resistance. With insulin resistance being a feature of PCOS as well as a comorbid factor of obesity, we investigated if PCOS affect their expression in adipose tissue directly and if the presence of obesity can cause a similar change or affect any PCOS induced alterations in the expression of these genes.

**Figure 3:**
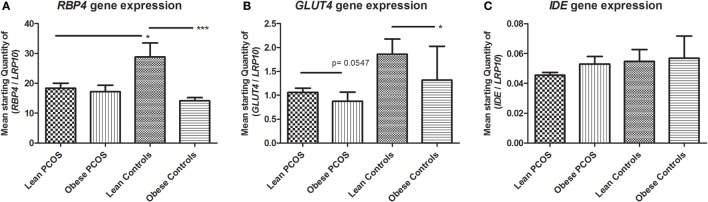
**Expression of markers of glucose metabolism in adipose tissue from lean and obese women with polycystic ovary syndrome (PCOS), and from lean and obese control women without PCOS**. Results are expressed as the mean starting quantity of (gene of interest/*LRP10*) ± SEM. **(A)** Expression of *RBP4*. **(B)** Expression of *GLUT4*. **(C)** Expression of *IDE*. *p < 0.05; ***p < 0.001. GLUT4, glucose transporter 4; LRP10, low-density lipoprotein receptor-related protein-10; RBP4, retinol-binding protein 4; and IDE, insulin degrading enzyme. Lean PCOS women, *n* = 17; obese PCOS women, *n* = 19; lean control women, *n* = 7; obese control women, *n* = 16. The groups were compared by two-way ANOVA analysis followed by subgroup analysis by Student’s *t*-test if significant. Level of statistical significance: 0.05.

A markedly higher *RBP4* expression was seen in lean controls compared to the other three groups which appeared similar (lean and obese PCOS women as well as obese controls) (Figure 3A). Supporting this, two-way ANOVA confirmed a significant interaction between weight and PCOS status (*p* = 0.0185), reflecting that PCOS status plays a role on *RPB4* expression and that this role depends on weight status, as no effect of PCOS *per se* was found (*p* = 0.1544, two-way ANOVA). In line with this, subgroup analysis using Student’s *t*-test showed a significant difference between lean PCOS and lean controls (*p* = 0.0170, *t*-test), but not between obese PCOS and obese controls (*p* = 0.4188), as a downregulatory effect similar to the one of PCOS was found of obesity (*p* = 0.0010, two-way ANOVA). According to this, a significant difference was seen when comparing obese controls vs. lean controls (*p* = 0.0002, *t*-test) whereas no difference was found when comparing obese PCOS women vs. lean PCOS women (*p* = 0.4286, *t*-test).

As shown by the two-way ANOVA analysis, the adipose expression of *GLUT4* was significantly downregulated by the presence of obesity (*p* = 0.0009), evident in subgroup analysis using Student’s *t*-test when comparing obese controls vs. lean controls (*p* = 0.0185, t-test) and tending toward statistically significance when comparing lean PCOS vs. obese PCOS (*p* = 0.0547, *t*-test). Two-way ANOVA analysis found no statistically significant effect of PCOS status on *GLUT4* expression (*p* = 0.1997) nor any statistical interaction between PCOS status and obesity (*p* = 0.1318), although *GLUT4* expression levels were higher in women in the lean control group than in any of the other three groups, as noted for *RBP4* (Figure 3B).

There were no statistical significant effects of PCOS status or obesity nor any interaction effect between the two on *IDE* expression levels by two-way ANOVA analysis (*p* = 0.9489, *p* = 0.6998 and *p* = 0.3055, respectively) (Figure 3C).

In summary, for *RBP4* expression, a high level was observed within the group of lean women compared to all other groups, which appeared similar, corresponding to a downregulatory response in lean PCOS women similar to the one observed when obese, and reflected as a statistically significant interaction between obesity and PCOS status. Likewise, expression of *GLUT4* was high in lean women compared to the other groups, indicating a potential effect of obesity and PCOS status, although only statistically significant of obesity on *GLUT4* expression.

## Discussion

Reduced levels of circulating zinc are common in obesity (8, 9) whereas recent studies report conflicting results with regard to the levels of circulating zinc in the blood of PCOS women (3–5). However, zinc supplementation has several beneficial effects on the metabolic status in PCOS women (6, 48). So far, limited attempts have been made to understand the underlying molecular events of the zinc disturbances in PCOS.

Investigating the expression of the zinc transporter *ZIP14* in adipose tissue, we found that *ZIP14* expression decreased in obese women with and without PCOS. Though, there was no statistically significant effect of PCOS itself on *ZIP14* expression. This correlation between the body weight and *ZIP14* expression supports our previous study of *ZIP14* in adipose tissue from otherwise healthy men and women, in which we found that *ZIP14* is downregulated in subcutaneous fat in response to obesity and that the downregulation is reversed by weight loss (27). Downregulation of *ZIP14* in a chronic condition with low-grade inflammation such as obesity might appear paradoxical, as murine studies have shown an upregulation of *Zip14* expression in liver, muscle, and adipose tissue after exposure to the pro-inflammatory lipopolysaccharide (29, 49). Studies in Zip14 knockout mice have linked lack of this specific transporter to inflammation, with evidence of endotoxemia and increased levels of circulating pro-inflammatory cytokines (29).

Moreover, Zip14 knockouts have increased amounts of body fat and display hypertrophic adipocytes with low differentiation potential in adipose tissues (29). Studies of 3T3-L1 pre-adipocytes confirm that Zip14 is associated with adipocyte differentiation (27–29), and that mobilization of intracellular zinc is decreased in adipocytes with downregulated *Zip14* expression (29).

The downregulation of Zip14 in adipocytes is speculated to result in a lack of free cytoplasmic zinc in adipocytes, defined as “the zinc trap” (29) by Troche and coworkers. The zinc trap refers to the fact that downregulation of Zip14 in 3T3-L1 adipocytes increases vesicular zinc yet reduces metallothionein expression (29). As metallothionein expression is tightly regulated by cytoplasmic zinc ions such low expression of metallothionein is considered indicative of a low concentration of free cytoplasmic zinc (50).

Although the present study employs fatty tissue samples as a whole—which includes macrophage involvement—our findings support a condition of cytoplasmic zinc deficiency within adipocytes in obesity, as we see downregulation of *ZIP14* paralleled by an upregulation of *ZNT1*, the only SLC30A efflux transporter in the plasma membrane (51). Supporting an obesity-related zinc dyshomeostasis, a reduced expression of *ZIP1–8* and *ZNT 2, 3, 6*, and *8* has previously been described in subcutaneous fat from obese individuals (52). However, such changes in zinc homeostasis are likely to be tissue specific. We recently reported decreased *ZNT1* and *ZNT6* expression in the human frontal cortex with increased BMI (53), and differences have been noted when comparing subcutaneous and visceral adipose tissues (52, 54). An altered expression of zinc transporters indicates a change in the pico-molar concentration and distribution of free intracellular zinc, but it is questionable if it will result in a measurable change in total zinc concentration within the range of a few 100 micro-molar. We have previously shown comparable levels of total zinc in adipose tissue from diabetic and non-diabetic sand rats on a high energy diet, as well as in blood samples from obese and non-obese individuals, despite changes in the expression profile of zinc transporters in adipose tissue (27, 54). Ultra-structural analysis of fresh tissue samples using the highly sensitive autometallographic technique might in the future provide valuable insights on the *in situ* location of free or loosely bound zinc (55); however, this method of silver amplification cannot be used for quantitative measurements.

The adipogenic transcription factor PPARG (56) links with PCOS, as specific PPARG gene polymorphisms associate with a reduced risk of PCOS and a better metabolic profile in PCOS women (31, 32). PPARG agonists are also used therapeutically in PCOS as it improves menstrual cyclicity, hormone levels, and glucose homeostasis (57). We observed that both obesity and PCOS reduce *PPARG* expression. Studies in adipocyte cultures, human biopsies, and tissues from Zip14 knockout mice have all shown a positive association between *PPARG* and *ZIP14* expression in adipose tissue (27, 29). Thus, Zip14 knockout mice display a decreased PPARG level and increased adipose tissue hypertrophy (29), indicating that the lack of free cytoplasmic zinc affects PPARG levels in these mice. Accordingly, free cytoplasmic zinc participates in cell differentiation by regulating different signaling pathways and transcription factors (58). Moreover, PPARG signaling and expression is decreased upon zinc deficiency and PPARG structurally requires zinc ions (33, 34). Therefore, it is likely that the *PPARG* downregulation occurring in obese individuals is caused by the decrease in *ZIP14* and upregulation of *ZNT1*. We found no synergistic effect between obesity and PCOS with regard to *PPARG* expression. In a larger study, however, it cannot be excluded that an association exists. *PPARG* expression was significantly reduced in lean women with PCOS, despite an unaltered *ZIP14* expression level, compared to lean controls, indicating that another signaling pathway could affect *PPARG* in PCOS. The high androgen level in PCOS women could account for some of the *PPARG* alterations, as androgens can decrease *PPARG* expression, as well as adipogenesis (59, 60).

Similar to *ZIP14, GLUT4* and *RBP4* expression are reduced in obesity. A statistically significant synergistic effect of PCOS and obesity was found for *RBP4*, and although not statistically significant for *GLUT4*, the lean controls appeared to be substantially different from both PCOS groups with regards to *GLUT4* expression. The obesity-related changes in these two genes could partly reflect a reduction in free cytoplasmic zinc. GLUT4 levels are downregulated in an obese insulin-resistant state (61) and regulated by the insulin signaling pathway (62), in which free intracellular zinc regulates several signaling steps (63). Zinc can also affect the transport/release of retinol and alter RBP4 levels in liver (64, 65). RBP4 has been speculated to be involved in inflammation and insulin resistance in obesity and type 2 diabetes (66), but the exact role and regulation in obesity is so far unclear (67). Increased RBP4 levels have been reported in adipose tissue from obese PCOS women vs. obese controls (68). In this study, *RBP4* expression was higher in the obese PCOS group vs. obese controls, although the change was not statistically significant with the present sample size.

ZIP14 seem to affect glucose metabolism as Zip14 knockout mice display hyperinsulinemia, augmented insulin receptor phosphorylation, and increased liver glucose (49, 69). ZIP14 might thus affect the symptomatology of PCOS *via* organs other than white fatty tissue. In this study, *ZIP14* expression in subcutaneous fat associates with markers of glucose metabolism in both blood and adipose tissue, although not as significantly as our previous findings in which the HOMA index, fasting glucose, and insulin correlated with *ZIP14* expression. However, our previous study included subjects of both sexes which had a higher average BMI in the obese group (27). As for *ZIP14*, the expression of the counter-regulated influx transporter *ZNT1* also showed significant correlations with markers of glucose homeostasis, further linking alterations in intracellular zinc to glucose metabolism.

Sex hormones regulate zinc transporters of the LIV-1 subfamily, and *ZIP14* expression can be induced by estrogen (26). However, we found no evidence that the specific hormonal changes occurring in PCOS affect *ZIP14* expression in adipose tissue. Furthermore, there were no changes in *ZIP9* expression, even though this transporter can function as an androgen receptor (37). Likely, obesity has a stronger influence on zinc homeostasis than the altered hormonal levels in PCOS.

In conclusion, PCOS is a complex disease that involves a zinc homeostatic disturbance and adipocyte dysfunction. The zinc transporters *ZNT1* and *ZIP14* show an inverse regulation in subcutaneous adipose tissue in obesity, as well as in obese women with PCOS. The downregulation of *ZIP14* expression and the upregulation of *ZNT1* expression in obese subjects are indicative of a reduction in free cytoplasmic zinc, with potential down-stream effects on *PPARG, RBP4*, and *GLUT4* expression, as well as *RBP4* appear to be directly involved in PCOS pathology.

## Author Contributions

TM, PS, AL, KS, and JL contributed to the measurements and analyses. KS, AL, JR, SP, and BB supervised TM when writing the manuscript and when designing the study. All the authors took part in the construction of the manuscript and have read and approved the final manuscript.

## Conflict of Interest Statement

The authors declare that they have no conflict of interest as the research was conducted in the absence of any commercial or financial relationships that could be construed as a potential conflict of interest.

## Abbreviations

BMI, body mass index; DHT, dihydrotestosterone; GLUT4, glucose transporter 4; HDL, high-density lipoprotein; HOMA index, homeostatic model assessment; IDE, insulin degrading enzyme; LDL, low-density lipoprotein; LRP10, low-density lipoprotein receptor-related protein-10; PCOS, polycystic ovary syndrome; PPARG, peroxisome proliferator-activated receptor gamma; RBP4, retinol-binding protein 4; SHBG, sex hormone-binding globulin; VLDL, very-low-density lipoprotein.
