# Supplementary material for: Expression Patterns and Correlations with Metabolic Markers of Zinc Transporters ZIP14 and ZNT1 in Obesity and Polycystic Ovary Syndrome
Source: Front Endocrinol (Lausanne). 2017 Mar 2;8:38. doi: 10.3389/fendo.2017.00038 (PMC5332389; doi:10.3389/fendo.2017.00038)
Supplement: Supplementary file 1 [file Table_1.DOCX]

Supplementary Material

**Expression Patterns and Correlations with Metabolic Markers of Zinc Transporters *ZIP14* and *ZNT1* in Obesity and Polycystic Ovary Syndrome**

**Trine Maxel*, Pernille Fog Svendsen, Kamille Smidt, Jesper Krogh Lauridsen, Birgitte Brock, Steen Bønlykke Pedersen, Jørgen Rungby, and Agnete Larsen**

*** Correspondence:** Trine Maxel: tmj@biomed.au.dk

| **Gene name (accession number)** | **Forward primer** | **Reverse primer** | **Annealing temperature**  **(°C)** |
| --- | --- | --- | --- |
| *GLUT4*  *(P14672)* | 5´­–CCCCATTCCTTGGTTCATCG–3´ | 5´–ATAGCCTCCGCAACATACTGG–3´ | 60 |
| *IDE*  *(P14735)* | 5´–GCCGAAGCCTTGTCTCAACT–3´ | 5´–CAAATAGGCCATGTTACAGTGCAA–3´ | 60 |
| *LRP10*  *(Q7Z4F1)* | 5´–TGCCATCCCACCTGTAGAAGAC–3´ | 5´–AGGTTGCCCAGCACTGAGTTATC–3´ | 60 |
| *PPARG1*  *(P37231)* | 5´–GTGGCCGCAGATTTGAAAGAA–3´ | 5´–CCATTACGGAGAGATCCACGG–3´ | 60 |
| *PPARG2*  *(P37231)* | 5´–GCAAACCCCTATTCCATGCT–3´ | 5´–ACGGAGCTGATCCCAAAGTT–3´ | 60 |
| *RBP4*  *(P02753)* | 5´–GACAACATCGTCGCGGAGTT–3´ | 5´–CCATGTCTGCGCACACGTCCC–3´ | 62 |
| *ZIP9 (Q9NUM3)* | 5´–TCGCTTGAGTCACTTACCC–3´ | 5´–TGCCAGTCATTAGATTCTCGT–3´ | 60 |
| *ZIP14*  *(Q15043)* | 5´–GCAGCAGCTGAAGGCCCTACT–3´ | 5´–CTCCCAATCCGCGACTG–3´ | 57 |
| *ZNT1*  *(Q9Y6M5)* | 5´–CGGAATTCACGCTACTACCAT–3´ | 5´–GGTTCTGCAGGCAAGTTCA–3´ | 60 |

**Supplementary Table 1.** Gene accession numbers and primer sequences used in the study. *Low-density lipoprotein receptor-related protein 10* (*LRP10*) was used as the housekeeping gene. *GLUT4*, *glucose transporter 4*; *IDE*, *insulin degrading enzyme*; *PPARG*, *peroxisome proliferator-activated receptor gamma*; *RBP4*, *retinol binding protein 4.*
